# Neuroimmunology of Behavioral Comorbidities Associated With Cancer and Cancer Treatments

**DOI:** 10.3389/fimmu.2018.01195

**Published:** 2018-06-07

**Authors:** Jessica C. Santos, Leah M. Pyter

**Affiliations:** ^1^Department of Basic and Applied Immunology, School of Medicine of Ribeirao Preto, University of Sao Paulo, Sao Paulo, Brazil; ^2^Departments of Psychiatry and Behavioral Health and Neuroscience, The Institute for Behavioral Medicine Research, Ohio State University Wexner Medical Center, Columbus, OH, United States

**Keywords:** depression, cognition, cytokines, neuroinflammation, neuropathic pain

## Abstract

Behavioral comorbidities (depression, anxiety, fatigue, cognitive disturbances, and neuropathic pain) are prevalent in cancer patients and survivors. These mental and neurological health issues reduce quality-of-life, which is a significant societal concern given the increasing rates of long-term survival after various cancers. Hypothesized causes of behavioral comorbidities with cancer include tumor biology, stress associated with the cancer experience, and cancer treatments. A relatively recent leading mechanism by which these causes contribute to changes in neurobiology that underlie behavior is inflammation. Indeed, both basic and clinical research indicates that peripheral inflammation leads to central inflammation and behavioral changes in other illness contexts. Given the limitations of assessing neuroimmunology in clinical populations, this review primarily synthesizes evidence of neuroimmune and neuroinflammatory changes due to two components of cancer (tumor biology and cancer treatments) that are associated with altered affective-like or cognitive behaviors in rodents. Specifically, alterations in microglia, neuroinflammation, and immune trafficking to the brain are compiled in models of tumors, chemotherapy, and/or radiation. Evidence-based neuronal mechanisms by which these neuroimmune changes may lead to changes in behavior are proposed. Finally, converging evidence in clinical cancer populations is discussed.

## Introduction

Over the past decade, advances in cancer diagnosis and therapy have increased the number of cancer survivors, substantially improving the relative percentages of 5-year survivors for the most common types of cancer in the United States (1). Regardless, cognitive impairments, fatigue, psychiatric comorbidities, and peripheral neuropathy, attributed largely to neurotoxic effects of cancer therapy, remain highly prevalent among cancer patients and survivors (2, 3). The cancer-related cognitive impairments are well-recognized and commonly referred to as “chemofog” or “chemobrain” [reviewed in Ref. (4)]. Indeed, intensity or duration of chemotherapy relates to the severity of chemobrain (5, 6), whereas psychological factors (e.g., depression) and surgery are largely independent (7). The cognitive domains most commonly implicated include learning and memory, concentration, executive function, and processing speed (8, 9), while the common psychiatric comorbidities include anxiety and depression (10). Cancer-related fatigue is characterized by persistent physical and mental tiredness that are not explained by recent activity and interfere with functional abilities [reviewed by Bower (11)]. Chemotherapy-induced peripheral neuropathy (CIPN) is another maladaptive and debilitating side effect of cancer treatment consisting of allodynia, hyperalgesia, and neuropathic pain, observed in 30–68% of patients and persisting even after completion of chemotherapy (12). Of note, fatigue strongly correlates more with pain than with cognitive impairments or mood in patients with cancer (13–15). Together, these behavioral symptoms are debilitating and reduce quality-of-life by limiting functional independence, reducing adherence to cancer treatment, undermining social and professional life, and generating a high psychosocial stress burden (16–18). They can manifest acutely or chronically, persisting in 35–75% of cancer patients for months or even years after they are cancer-free (19–21). Such large discrepancies in prevalence are likely related to differences in cancer types and treatments or methodological assessments across studies. However, the biological mechanisms underlying these comorbidities remain unclear. Therefore, preventative approaches for behavioral changes have not been standardized and effective treatment remains a serious clinical problem (22). Substantial evidence has associated cancer treatment, especially chemotherapy, with brain damage and these behavioral comorbidities. The mechanisms by which chemotherapy induces brain neurotoxicity are hypothesized to involve neuroinflammation, damage associated molecular patterns (DAMPs) (23), impaired neurogenesis (24–32), oxidative stress, myelin degradation, and blood–brain barrier (BBB) degradation (33). Similarly, CIPN involves peripheral neuron damage and axonal degeneration (34, 35) *via* neuroinflammatory mechanisms in the spinal cord (34, 36–42).

Radiation therapy directed at the brain also has obvious effects on behavior and neuroimmunology (43, 44), whereas radiation directed outside of the brain was long considered localized and, therefore, without consequences on the brain. However, recent evidence in various cancer populations indicates that radiation directed away from the brain still induces fatigue, as well as executive function and memory problems that persist for years after therapy (45–47), potentially through the actions of radiation-induced bystander effects (e.g., inflammation).

In addition to cancer treatments, numerous studies demonstrate that tumor biology by itself is able to influence neurocognitive function and affect. For example, behavioral impairments are observed in treatment-free, tumor-bearing mice (30, 48–56) and in cancer patients before they start chemotherapy (57–69). Tumorigenesis is a complex and multistep process, consisting of tumor initiation, progression, and dissemination. The solid tumor microenvironment contains various non-tumor cell populations such as endothelial, stromal, and innate inflammatory immune cells that support tumor progression (70). Thus, peripheral-to-central inflammation has been implicated as a key pathway underlying these tumor-induced changes in behavior. In addition, tumors can affect endocrine stress pathways, thereby indirectly modulating neuroimmunology and behavior [reviewed by Pyter (71)]. This review will focus on the recent and expanding primary literature supporting a role for innate immunity and inflammation in tumor- and cancer treatment-induced behavioral symptoms.

Indeed, innate immune cell activation within the central nervous system (CNS) is a key factor driving neuroinflammation, with resident microglial cells as the primary cellular venue (72). Pattern recognition receptors, such as toll-like receptors (TLRs) and NOD-like receptors (NLRs), are constitutively expressed by microglia, astrocytes, and oligodendrocytes in the brain. These receptors recognize pathogen associated molecular patterns and DAMPs, which are “sterile” inflammatory signals released by dying cells in the periphery or brain (73). TLR activation elicits canonical NF-κB signaling, whereas NLR activation induces the assembly and activation of inflammasomes (multiprotein cytosolic complexes), each of which trigger pro-inflammatory caspases to cleave the pro-inflammatory cytokines, interleukin (IL)-1β, IL-18, and IL-33, into their active forms (74). Mounting evidence implicates microglial activation and its associated neuroinflammation in the pathogenesis of multiple neurological and psychiatric disorders, such as depression, Alzheimer’s disease, multiple sclerosis, cognitive impairments, and normal aging (75–81). In terms of these chronic peripheral inflammatory conditions, basic science data indicate that cytokines can stimulate peripheral nerves (e.g., vagus) and/or humorally transduce inflammatory signals into the CNS and drive behavioral changes (82). In addition, recent studies indicate that TBI, stroke, and experimental autoimmune encephalomyelitis (multiple sclerosis model) increase BBB permeability (83–85), allowing inflammatory mediators and peripheral immune cells to directly enter the brain. Thus, it is possible that tumors or cancer treatments may also influence brain function by altering innate immune cell trafficking directly to the brain.

The pathway between cancer and the CNS is hypothesized to be bidirectional. Indeed, the concept that depression or stress may precipitate chronic inflammatory diseases, including cancer (86), has existed for centuries and has been reviewed elsewhere (87). Here, we focus on one direction of this bidirectional relationship: the tumor- and tumor treatment-induced neuroinflammation contributing to affective-like, pain, and cognitive behaviors. Cancer-related fatigue and its underlying immune mechanisms are thoroughly reviewed elsewhere (11). Understanding how tumor biology and cancer treatments can interact to lead to changes in the brain will allow for improved targeting by therapeutic interventions focused on behavioral issues and thereby increase quality-of-life and survival for cancer patients. Although behavioral comorbidities are relevant for both brain and peripheral tumor patients, brain tumors and their treatments impact the brain much more directly than peripheral tumors. Indeed, brain tumor effects on behavior are confounded by the fact that they physically disrupt the brain/brain immune system and their treatments directly target brain tissue; therefore, only tumors outside of the brain will be discussed here. It is important to note that despite relatively consistent behavioral issues reported among some cancer patients, their tumors, and cancer treatments are heterogeneous and complex. Finally, we focus on the most common cancer treatments of chemotherapy and radiation, however, cancer patients are also treated with other anticancer (e.g., immunotherapy), anti-nausea, anti-infection drugs, which likely further contribute to mental health issues.

## Rodent Models of Cancer, Neuroimmunology and Behavior

Current basic research using rodent cancer models implicates several putative mechanisms underlying behavioral changes. These non-human models allow for a more neurobiological understanding of the effects of tumors and tumor treatments on behavior compared to clinical research. They can also elucidate the effects of specific cancer therapies by themselves by using tumor-free mice, thereby simplifying the complex interactions between tumors and multiple tumor treatments inherent to clinical populations.

The methods for identifying current reports in the English language on how cancer and cancer treatments drive behavioral and/or neuroimmune changes in rodent models consisted of PubMed searches through April 2018 using combinations of the MeSH search terms: (“rodent”; “cancer” or “neoplasms, experimental,” or “tumor”; “inflammation” or “cytokine” or “microglia” or “neuroinflammation”; “behavior” or “cognition” or “learning” or “affect” or “depression” or “anxiety”; “chemotherapy” or “chemobrain” or “radiation” or “neuropathy” or “neuropathic pain”). Notably, only tumor models consisting of tumors located outside of the brain were considered. Here, we present tumor-bearing models with and without cancer treatments, followed by tumor-free models with cancer treatments.

### Neuroimmunology in Tumor-Bearing Rodent Models

**Figure 1:**
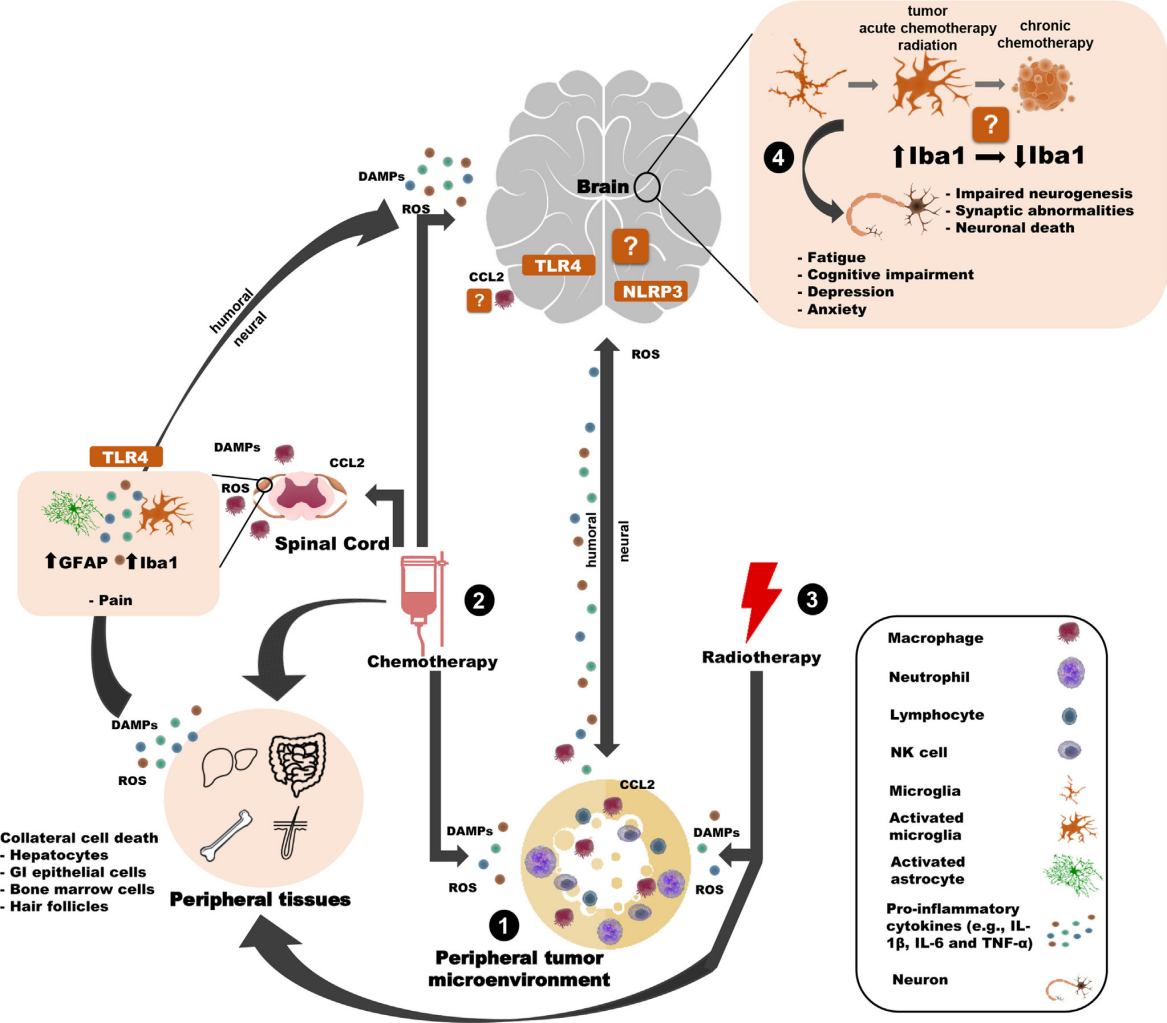
Potential innate immune mechanisms by which peripheral cancer and cancer treatments can induce behavioral changes. (1) The tumor microenvironment releases pro-inflammatory mediators (e.g, cytokines) that can influence the brain and behavior through humoral or neural routes. (2) Chemotherapy induces cell death of tumor cells and healthy cells (in the brain and the periphery), thereby causing the release of DAMPs, ROS, cytokines, and chemokines and contributing to many side effects. For example, chemotherapy-induced peripheral neuropathy is associated with astroglial and microglial activation in the spinal cord and TLR4 activation in DRG neurons. Similar inflammasome activity may occur in the brain. Chemotherapy may also weaken the blood–brain barrier, allowing peripheral immune cells to traffic into/closer to the brain. (3) Peripheral radiotherapy induces cell death of tumor cells and healthy “bystander” cells and (indirectly) contributes to microglial activation and behavioral deficits. (4) Together, the tumor and cancer treatments influence microglia. Tumors and radiotherapy (indirectly) activate microglia, whereas chemotherapy may affect microglia differently over time. Microglia interface with neurons to affect behavior, potentially through. Certain elements of this work were taken and then adapted from somersault18:24 (Library of Science & Medical Illustrations). To view their site, visit http://www.somersault1824.com/. They are licensed under the Creative Commons Attribution-NonCommercial-ShareAlike 4.0 International License. To view a copy of this license, visit http://creativecommons.org/licenses/by-nc-sa/4.0/ or send a letter to Creative Commons, PO Box 1866, Mountain View, CA 94042, USA.

In solid peripheral neoplasms, tumor and non-tumor cells in the tumor microenvironment (e.g., leukocytes, fibroblasts, endothelial cells) secrete inflammatory mediators that attract additional immune cells, and promote tumor growth, development, and metastasis (70, 88, 89). Among the most common inflammatory mediators increased by tumors are cytokines and chemokines, including IL-1, TNF-α, IL-6, IL-8, IFN-α, IL-10, IL-12, TGF-β, and CXCR4 (90, 91). These inflammatory mediators are released into circulation and can be transduced into the brain potentially *via* neural and humoral pathways (92) leading to neuroinflammation, which in turn influences behavior (89) (Figure 1). Of note, increases in circulating cytokines are detectable only in some tumor models and during specific stages of the tumor development (89), although these humoral elevations are not mandatory to induce neuroinflammation and behavioral alterations (82).

Our previous review focuses on the behavioral consequences of tumors in rodents without cancer treatments (89). Several of these behavioral studies also report concomitant tumor-induced immune changes in the brain and/or in the periphery. For example, brain pro-inflammatory cytokines (IL-1β, IL-6, and TNF-α), as well as inflammatory enzymes and signaling factors [nitric oxide synthase (iNOS), indolamine 2,3-deoxygenase, cyclooxygenase-2 (COX-2)], increase along with affective-like behavior, fatigue, or cognitive impairments, when various solid tumors are generated in the periphery [(30, 48, 50, 52, 54–56), but see Ref. (93)]. Likewise, circulating pro-inflammatory cytokine increases are frequently observed in solid tumor models [(30, 51, 53–55, 93, 94), but see Ref. (48, 51)]. These inflammatory changes are hypothesized to drive the accompanying behavioral changes, however, rarely are statistical relationships between the two assessed. Of the reports statistically linking behavior and inflammation, our lab and others demonstrate that circulating cytokines (51), tumor mass, and/or tumor-derived cytokine gene expression are positively associated with neuroinflammation, fatigue, or anxiety-like behavior in female mice with peripheral tumors (94). Indeed, the consistent increase in brain IL-1β levels in tumor-bearing mice (50, 55, 56, 95) suggests a putative role for inflammasomes not only during chemotherapy, but also in cancer-induced depressive-like behavior. However, a different mammary tumor model reports neuroinflammation, but in the absence of affective-like behavior, cognitive deficits, or changes in neurogenesis (93). These discrepancies in behavioral and neurobiological outcomes might be due to differing methodological approaches. While microglial activation is well-established in brain tumor models (96), recent evidence indicates that brain microglia may be the cellular source of this neuroinflammation in various peripheral tumor models, as is observed through increased ionized calcium-binding adaptor molecule 1 (Iba1) immunoreactivity or *Cd11b* gene expression in the cortex and hippocampus (brain regions that regulate affect, energy, and cognition) (55, 56, 94, 95) (Table 1). Similarly, microglial activation in the spinal cord is associated with bone pain in bone cancer models (34).

**Table 1:** Summary of cancer- and cancer treatment-induced neuroinflammatory changes in rodents.

Reference	Treatment	Tumor	Behavioral effects	Central inflammatory measures
Pyter et al. (48)	No treatment	Rat mammary tumor	Depressive- and anxiety-like behaviors	↑ IL-1β, IL-6, TNF-α, and IL-10 (Hi)

Pyter et al. (50)	No treatment	Rat mammary tumor	Cognitive impairment	↑ IL-1β (Hi)

Pyter et al. (95)	No treatment	Rat mammary tumor	Impaired recovery from sickness behavior	↑ CD11b and IL-1β (Hi—basal conditions)
				↑ CD11b (Hi and Ctx—4 h after immune challenge)
				↑ IL-1β (Hi and Ctx—4 h after immune challenge)

Lebeña et al. (54)	No treatment	Mouse melanoma	Depressive-like behavior	↑ IL-6 and TNF-α (Hi)

Yang et al. (30)	No treatment	Mouse colorectal	Depressive-like behavior and cognitive impairment	↑ IL-6 and TNF-α (Hi)
				↓ COX-2 (Hi)

Norden et al. (55)	No treatment	Mouse colorectal	Depressive-like behavior (anhedonia)	↑ Microglia activation (Iba1^+^ cells—Ctx)
				↑ IL-1β (Hi and Ctx) and IL-6 (Ctx)

Norden et al. (226)	No treatment	Mouse colorectal	Depressive- and fatigue-like behavior(prevented by ibuprofen)	↑ IL-1β and IL-6 (Hi; reduced by ibuprofen)

Walker et al. (93)	No treatment	Mouse metastatic mammary tumor	N/C	↑ IL-1β (Hi and Ctx)
	
		Mouse non-metastatic mammary tumor		N/C

Vichaya et al. (56)	No treatment	Mouse human papilloma virus-related neck and head cancer	N/C	↑ CD11b and TNF-α (Hyp—basal conditions)
				↑ IL-1β (Hi, Hyp, Ctx, CS, and Cb)
	
			Impaired motivated behavior, locomotor activity and depressive-like behavior 24 h after immune challenge	↑ IL-6 (Cb—24 h after immune challenge)

Pyter et al. (94)	No treatment	Mouse mammary tumor	Anxiety-like behavior	↑ CD11b and CXCL1 (Hi)
		
		Mouse mammary tumor resected	Tumor resection exacerbated anxiety-like behavior	Tumor resection reversed hippocampal CD11b and CXCL1 increase and ↑ cortical CXCL1

Seigers et al. (118)	Methotrexate	Tumor-free rat	N/A	↑ Microglia activation (Iba1^+^ cells—Hi) and N/C in cytokines (Hi) or [11C]PK11195 uptake

Seigers et al. (113)	Cyclophosphamide	Tumor-free mouse	N/A	↓ Microglia (Iba1^+^ cells—Ctx)
	
	Docetaxel			↓ Microglia (Iba1^+^ cells—Ctx)
	
	Doxorubicin			N/C
	
	5-fluorouracil			↓ Microglia (Iba1^+^ cells—Ctx)
	
	Methotrexate			N/C
	
	Topotecan			↓ Microglia (Iba1^+^ cells—Ctx)

Christie et al. (29)	Cyclophosphamide	Athymic tumor-free nude rat	Cognitive impairment	↑ Microglia activation (ED1^+^ cells—Hi)
	
	Doxorubicin			N/C

Paquet et al. (112)	Paclitaxel	Breast cancer xenograft in nude mouse	N/A	N/C
		
	Epirubicin + Cyclophosphamide	Breast cancer xenograft in nude mouse		↓ Microglia (Iba1^+^ cells—Hi, CS, Ctx, and Cb) N/C
		
	Paclitaxel	Tumor-free nude mouse		N/C
		
	Epirubicin + Cyclophosphamide	Tumor-free nude mouse		↓ Microglia (Iba1^+^ cells load—Hi, CS, Ctx, and Cb)

Yang, et al. (52)	Methotrexate	Mouse mammary carcinoma	Cognitive impairment and depressive- like behavior	↑ iNOS and COX-2 (Hi)
				↑ Microglia (Iba1—Hi)

Salas-Ramirez et al. (117)	Doxorubicin + Cyclophosphamide	Tumor-free rat	Cognitive impairment	↑ Erk1/2 and Akt activation in OVX female rats (Hi)

Zhang et al. (129)	Paclitaxel	Tumor-free rat	Neuropathic pain	↑ Astrocytes activation (GFAP^+^ cells in the spinal cord)

Zhang et al. (38)	Paclitaxel	Tumor-free rat	Neuropathic pain (attenuated by anti-CCL2 treatment)	↑ CCL2 (spinal astrocytes)

Pevida et al. (40)	Paclitaxel	Tumor-free mouse	Neuropathic pain (prevented by anti- CCL2 or minocycline treatment)	↑ CCL2 (lumbar spinal cord)
				↑ Microglia (Iba1^+^ cells in the lumbar spinal cord)

Ruiz-Medina et al. (126)	Paclitaxel	Tumor-free mouse	Neuropathic pain	↑ Microglia and astrocytes (Iba1^+^ and GFAP^+^ cells in the spinal cord)

Mannelli et al. (128)	Oxaliplatin	Tumor-free rat	Neuropathic pain (prevented by pharmacological microglia or astrocyte inhibition)	↑ Microglia and astrocytes activation (Iba1^+^ and GFAP^+^ cells in the dorsal horn)

Huang et al. (39)	Paclitaxel	Tumor-free rat	Neuropathic pain (attenuated by anti-CX3CL1 treatment)	↑ CX3CL1 and caspase-3 (A-fiber primary sensory neurons)

				↑ Macrophages infiltration (DRG; prevented by anti-CX3CL1 treatment)

Li et al. (37)	Paclitaxel	Tumor-free rat	Neuropathic pain (transiently reversed by TLR4 antagonist treatment)	↑ TLR4 (spinal astrocytes and DRG neurons), MyD88, and TRIF (DRG neurons)

Li et al. (36)	Paclitaxel	Tumor-free rat	Neuropathic pain (prevented by MAPK inhibitors)	↑ pERK1/2 and pP38 (DRG)
				↑ TLR4 signaling *via* MAP kinases and NF-κB (DRG)

Zhang et al. (42)	Paclitaxel	Tumor-free rat	Neuropathic pain (reduced by intrathecal TLR4 antagonist treatment, CCL2 neutralization or macrophage depletion)	↑ TLR4 activation, CCL2 expression, and macrophages infiltration (DRG—reduced by intrathecal TLR4 antagonist treatment or CCL2 neutralization)

Makker et al. (41)	Oxaliplatin	Tumor-free mouse	Neuropathic pain	↓ Microglia (P2ry12^+^ cells—dorsal/ventral horns)
	
	Paclitaxel			↓ Microglia (P2ry12^+^ cells—dorsal/ventral horns)
	
				↑ TNF-α, IFN-γ, CCL11, CCL4, CCL3, IL-12p70, and GM-CSF (spinal cord)

Jia et al. (147)	Paclitaxel	Tumor-free rat	Neuropathic pain (alleviated by a non-specific ROS scavenger)	↑ NLRP3, caspase-1, and IL-1β (DRG) reversed by a non-specific ROS scavenger

				↑ NLRP3 in CD68^+^ macrophages and (DRG and sciatic nerve)

				Mitochondrial damage (spinal cord)

Ledeboer et al. (127)	Paclitaxel	Tumor-free rat	Neuropathic pain (attenuated by intrathecal IL-1 receptor antagonist or IL-10 gene therapy)	↑ Microglia activation (OX-42 and OX-6^+^ cells in the spinal cord)
				↑ CD11b, TNF-α, and IL-1β (DRG), attenuated by intrathecal IL-10 gene therapy

Hu et al. (227)	Cisplatin	Tumor-free mice	Neuropathic pain (attenuated by minocycline or anti-TREM2 treatment)	↑ TREM-2-mediated microglia activation (Iba1^+^ cells in the spinal cord)
				N/C in astrocyte activation
				↑ IL-6, TNF-α, IL-1β, iNOS, and CD16 (spinal dorsal horn—attenuated by intrathecal minocycline) and TREM-2 (spinal cord)

McGinnis et al. (9)	Radiotherapy + Anti-CTLA-4	Tumor-free BALB/c mouse	↓ Anxiety-like behavior (in some cases) and ↑ Cognitive impairment (in all cases)	↑ Microglia activation (CD68^+^ cells—Ctx and Hi)
		Tumor-free C57BL/6J mouse		
		BALB/c mouse colorectal		
		C57BL/6J mouse lung carcinoma		

Feiock et al. (21)	Radiotherapy	Tumor-free mouse	N/A	↑ Microglia (Iba1^+^ cells—Hi, CS, Ctx, and Cb) and astrocyte (GFAP^+^ cells—CS, Ctx, and Cb) activation and TNF-α (Hi)
	Methotrexate			

Acharya et al. (123)	Cyclophosphamide	Athymic tumor-free nude rat	Cognitive impairment (ameliorated by stem cell transplantation treatment)	↑ Microglia activation (CD68^+^ cells—Hi) reversed by stem cells transplantation treatment

Cheruku et al. (229)	Doxorubicin	Tumor-free rat	Cognitive impairment (ameliorated by Catechin treatment)	↑ MPO levels (Hi and Ctx) reversed by Catechin treatment

El-agamy et al. (230)	Doxorubicin	Tumor-free rat	Cognitive impairment ameliorated by Astaxanthin treatment	↑ TNF-α, PGE_2_, and COX-2 levels (Hi) and astrocytes activation (GFAP^+^ cells) reversed by astaxanthin treatment

Ramalingayya et al. (231)	Doxorubicin	Tumor-free rat	Cognitive impairment ameliorated by Rutin treatment	↑ TNF-α levels (Hi and Ctx) reversed by astaxanthin treatment

*IL-1β, interleukin-1 beta; IL-6, interleukin-6; TNF-α, tumor necrosis factor alpha; IL-10, interleukin-10; Cd11b, cluster of differentiation molecule 11b; COX-2, cyclooxygenase-2; Iba1, ionized calcium-binding adaptor molecule 1; CXCL1, C-X-C motif chemokine ligand 1; [11C]PK11195, (1-(2-chlorophenyl)-N-methyl-N-(1-methylpropyl)-3-isoquinoline carboxamide); ED-1, anti-CD68; CD68, cluster of differentiation 68; iNOS, inducible nitric oxide synthase; ERK1/2, extracellular signal-regulated protein kinases 1 and 2; Akt, protein kinase B; OVX, ovariectomized; GFAP, glial fibrillary acidic protein; CCL2, C–C motif chemokine ligand 2; CX3CL1, C-X3-C motif chemokine ligand 1; TLR4, toll-like receptor 4; Myd88, myeloid differentiation primary response 88; TRIF, TIR-domain-containing adapter-inducing interferon-β; pERK1/2, phospho-extracellular signal-regulated protein kinases 1 and 2; pP38, phospho-P38; MAP, mitogen-activated protein kinase; NF-κB, nuclear factor-κB; P2ry12, purinergic receptor P2Y; INF-γ, interferon gamma; CCL11, C–C motif chemokine ligand 11; CCL4, C–C motif chemokine ligand 4; CCL3, C-C motif chemokine ligand 3; IL-12p70, interleukin-12p70; GM-CSF, granulocyte-macrophage colony-stimulating factor; NLRP3, nucleotide-binding domain, leucine-rich-containing family, pyrin domain-containing-3; OX-42, anti-complement type 3 receptors; OX-6, anti-major histocompatibility complex class II; TREM-2, triggering receptor expressed on myeloid cells 2; CD16, surface Fcγ receptor; MPO, myeloperoxidase; PGE2, Prostaglandin E2; Hi, hippocampus; Hyp, hypothalamus; CS, corpus striatum; Ctx, cortex; Cb, cerebellum; DRG, dorsal root ganglion; N/C, no change; N/A, not applicable*.

Furthermore, elevations in *Cd11b* and other neuroinflammatory mediators, as well depressive-like and sickness behaviors, are attenuated by minocycline anti-inflammatory treatment in murine models of colon cancer and human papilloma virus (HPV)-related neck and head cancer (55, 56). As further evidence that tumors appear to be causal, and perhaps have long-lasting consequences on microglial-related changes, complete surgical resection of non-metastatic mammary tumors partially reverses tumor-induced neuroinflammation and circulating cytokines, but amplifies anxiety-like behavior (94).

Although hippocampal microglial activation (*Cd11b* expression) at rest is consistent among the majority of these tumor models, the results concerning regional expression of *Cd11b* mRNA in the brain to a subsequent peripheral immune challenge are mixed. One such challenge, lipopolysaccharide (LPS) injection (i.p.) increases cortical and hippocampal *Cd11b* expression, but decreases its expression in the hypothalamus of mammary tumor-bearing rats (95). Moreover, in HPV-related neck and head tumor-bearing mice, LPS does not change hippocampal and cortical *Cd11b* expression (56). Such discrepancies in the neuroinflammatory response are likely related to differences in cancer types and LPS doses. Taken together, these results indicate that baseline inflammation and neuroinflammatory responses to secondary immune challenges are influenced by tumors, although the identification of specific underlying mechanisms requires further investigation.

While microglial cells are the primary drivers of neuroinflammation within the CNS, increasing evidence suggests that other neuroimmune mechanisms are associated with behavioral changes. For example, psychological stress induces myeloid-derived cell trafficking to the brain, which in turn, induces affective-like behavior (81). While this review focuses on cancer and cancer treatments, it is important to note that stress associated with a cancer diagnosis may exacerbate tumor inflammatory activation, increasing tumor burden and development of metastases (97–100), and leading to more severe behavioral symptoms (51, 101, 102) through similar neuroimmune mechanisms. Furthermore, bone marrow-derived monocytes, including perivascular cells, meningeal macrophages, dendritic cells, and monocytes, have been implicated in the brain innate response in several neurologic and psychiatric diseases, as well as peripheral acute infections with sickness behavior (103–105). Indeed, in addition to potential humoral and neural routes by which peripheral inflammation is transduced into neuroinflammation, tumors affect immune trafficking to various areas of the body (94). Thus, immune trafficking of circulating monocytes to the brain may also play a role in tumor-induced changes in neurobiology and behavior (106–108) and warrants investigation.

### Neuroimmunology and Cancer Treatments in Tumor-Bearing Rodents

Chemotherapy is a common adjuvant cancer treatment (109). While most basic science reports focus separately on either tumors or chemotherapy, a few combine the two for a more clinically-relevant (albeit complex) model. The combination of tumors and chemotherapy could additively increase peripheral inflammation or potential BBB disruption, thereby allowing peripheral inflammatory mediators to reach the brain, induce neurotoxicity and neuroinflammation, and contribute to cognitive and affective symptoms (8, 25, 52).

Both human and non-human research suggests that cancer treatment is causally related to the development of mood and anxiety disorders, although the potential underlying mechanisms remain broad. For example, antimetabolite chemotherapy (methotrexate) induces significant depressive-like behavior and cognitive impairments associated with an upregulation of pro-inflammatory enzymes (iNOS and COX-2) and activation of microglia in the brains of mammary tumor-bearing mice (52). In contrast, methotrexate suppresses peripheral cytokine levels in other studies (110, 111). Combined epirubicin and cyclophosphamide chemotherapies reduce stereological hippocampal microglial Iba-1 expression in the hippocampus, cortex, striatum, and cerebellum of both tumor-free and nude mice xenografted with patients’ tumor samples compared to xenografted mice treated with saline (112). These Iba1 reductions may represent chemotherapy-induced microglial cell death (113). Behavioral changes were not assessed in this study. Taken together, the mixed inflammatory results from tumor-bearing models treated with chemotherapy indicate that tumor-free models are still necessary to clarify the individual roles of chemotherapeutic agents and tumors in associated behavioral impairments.

Emerging basic and clinical research indicates that stress and cancer treatments interact to influence tumor-associated immune and behavioral symptoms (101, 114). For example, physical restraint stress in tumor-bearing mice treated with cyclophosphamide impairs the antitumoral immune response, thereby reducing the therapeutic effects of the chemotherapy treatment (115). The potential synergistic effects of stress on cancer treatment-induced neuroinflammation remain to be determined. Finally, limited data are available on radiation as another potential cancer treatment contributor to neuroinflammation and/or behavioral consequences in tumor-bearing rodent models. One recent study evaluated these changes in tumor-free and tumor-bearing mice that received peripheral radiotherapy or immunotherapy (anti-CTLA-4 antibody) or both. Of note, the mice received precise peripheral irradiation to the tumor site in the right flank. Immunotherapy alone or in combination with radiotherapy induces cognitive impairments, increases in CD68^+^ microglial immunostaining and central cytokine production (9). Thus, the current literature concerning neuroinflammatory-dependent behavioral changes in rodent cancer models indicates that a variety of cancer treatments are likely relevant, despite their different mechanisms of action.

### Neuroimmunology and Cancer Treatments in Tumor-Free Rodent Models

The most extensive investigation regarding the potential mechanisms by which cancer treatments alter behavior is derived from studies using chemotherapeutic agents in tumor-free rodent models. Within this literature, some reports focus on behavioral consequences, neuroimmune consequences, or both. Notably, the reported behavioral effects vary based upon the particular agents and administration paradigms used, as well as the specific behavioral tests employed. The short- and long-term behavioral changes following chemotherapy treatment predominantly consist of impaired performance in learning and memory tasks including reference and working spatial performance, novel object recognition, and object placement without affecting general motor function (113, 116, 117). The majority of these studies report generalized hippocampal and cortical cellular or myelin (protective sheath of neuronal axons) damage in the brain, with some evidence of microglial cell death (25, 29, 118–120). Biochemical testing of some chemotherapeutic agents indicates that they should not be able to cross the BBB (121), alternatively suggesting that chemotherapy metabolites or other indirect mechanisms, such as peripheral immune cell infiltration, may be driving these neurobiological consequences (25). How neuroinflammation can alter neuronal function to cause behavioral changes is discussed in section “Link Between Neuroimmunology and the Neuroscience of Behavior.”

Several reports indicate a role for neuroimmune activation in chemotherapy-induced behavioral deficits (Table 1). For example, methotrexate induces microglia activation (Iba-1^+^ staining) in the hippocampus 1 and 3 weeks after treatment in tumor-free rats, in addition to reducing hippocampal blood vessel density (122). However, the peripheral cytokine levels and positron emission tomography scans for the uptake of [11C]PK11195 (a marker that has been associated with neuroinflammation and increased microglia activation) do not support neuroinflammatory changes underlying to immunohistochemistry results. Similarly, clinically-relevant dosing of chronic cyclophosphamide and doxorubicin treatments in tumor-free rats induces impairments in hippocampal-based memory and reduces neurogenesis (29). Coincident with the behavioral and neurogenesis changes, cyclophosphamide, but not doxorubicin, induces microglia activation (ED-1^+^ staining). In another report, impairments in different cognitive domains were observed in tumor-free mice after cyclophosphamide, docetaxel, doxorubicin, 5-fluorouracil, methotrexate, or topotecan treatment (116), while a reduced number of microglial cells (Iba-1^+^ cells) were observed in the prefrontal cortex for all these treatments (three weeks after treatment) compared with control mice, except methotrexate and doxorubicin treatment (113). In addition, chronic cyclophosphamide treatment in athymic nude rats induces microglial activation (increased CD68^+^ cells) in the hippocampus, as well as cognitive impairments in hippocampal and cortical-dependent tasks (123). These mixed microglial results indicate that multiple chemotherapeutic mechanisms of action may converge to trigger neuroinflammation and behavioral changes and that neuroinflammation may be due to microglial activation, microglial cell (or other cell) death or even disruptions in microglial homeostasis. Interestingly, some studies have reported a correlation between chemotherapy-induced circulating and central pro-inflammatory cytokine (IL-6, TNF-α, and IL-1β) concentration and behavioral changes acutely, but not chronically, suggesting that different mechanisms might be driving the initiation and the persistence of these comorbidities (124, 125).

Several chemotherapeutic drugs, such as platinum-based drugs (cisplatin, oxaliplatin), vinca alkaloids (vincristine), and taxanes (paclitaxel and docetaxel), trigger CIPN [as reviewed by Starobova and Vetter (35)] that is associated with neuroinflammation, although the literature is rather conflicting. Some studies indicate a key role for microglial activation [increased Iba-1, OX-42 (complement type 3 receptors), OX-6 (major histocompatibility complex class II) immunoreactivity, and *Cd11b* gene expression] in the spinal cord, or specifically, the dorsal root ganglion (DRG) of sensory neurons within the spinal cord (40, 126, 127). This microglial activation can be reversed by minocycline antibiotic treatment (40, 128) or intrathecal anti-inflammatory IL-10 gene therapy (127). However, the majority of CIPN studies implicate astrocyte activation (increased GFAP immunoreactivity and astrocyte hypertrophy) (36, 37, 41, 126, 128, 129). With CIPN, paracrine activation of CCL2/CCR2 signaling occurs and/or increased levels of CX3CL1 drive immune trafficking of activated macrophages to DRG, inducing nerve damage (39), which can be inhibited by anti-CCL2 antibody treatments or macrophage depletion (38–40, 42). Moreover, recent studies reported an increase in TLR4 signaling in spinal cord astrocytes and neurons in the DRG (36, 37). Potential pathways by which paclitaxel chemotherapy contributes to CIPN *via* TLR4 activation are the downstream canonical (myeloid differentiation primary response gene 88) and non-canonical pathways (TIR-domain-containing adapter-inducing interferon-β), culminating in NF-κB activation and upregulation of pro-inflammatory cytokines and chemokines including TNF-α, IFN-γ, IL-6, and CCL2 (36, 37, 41). TLR4 activation has been also associated with the sensitization of the ionic channel transient receptor potential vanilloid subtype 1 (TRPV1) (36, 130), found in nociceptors. Oxaliplatin chemotherapy also induces CIPN by upregulating pro-inflammatory cytokines and chemokines (IL1-β, TNF-α, IL-6, IL-8, and CCL2), which sensitizes nociceptors. The adaptive immune system is also likely involved in these responses as both paclitaxel and oxaliplatin increase the circulating levels of CD4^+^ and CD8^+^ T-cells (41). In addition, one study (131) indicates that the NLRP3 inflammasome pathway may also be activated *in vitro* in primed murine bone marrow-derived macrophage during anthracycline-induced IL-1β release. This suggests that some of the associated side effects, including behavioral changes, may be attenuated by IL-1β suppression. Indeed, intrathecal injection of IL-1ra transiently reversed paclitaxel-induced allodynia (127). NLRP3 inflammasome activation is driven by mitochondrial damage and reactive oxygen species (ROS) production in infiltrated macrophages of DRG and peripheral nerves and is also thought to play a role in paclitaxel-induced CIPN. Of note, some studies implicate neurotoxic effects of antineoplastic agents, which impair axonal trafficking leading to myelin and axon damage in CIPN, suggesting that the cellular damage may precede the neuroinflammation in the DRG [reviewed by Nicolini et al. (132)].

Finally, the induction of biological consequences on cells that are not directly transected by radiation treatment due to the signaling of those cells that are, is termed radiation-induced bystander effects. Both *in vitro* and *in vivo* models demonstrate off-target consequences of radiation on epigenetics, DNA health, apoptosis, cell proliferation, tumorigenesis, and inflammation (133). Indeed, peripheral radiation treatment to the right hind limb in tumor-free mice increases microglial Iba1^+^ cell numbers and TNF-α gene expression in the brain, comparable to the neuroinflammation observed following chemotherapy treatment (21). Whole-body radiation-induced neuroinflammation is associated with pro-inflammatory gene expression and reduced locomotion (134), although the direct brain radiation may be responsible for these effects. Radiation in rodents is also associated with general increases in circulating inflammation (135, 136), which coincide with fatigue (i.e., reduced locomotion) (137, 138). Despite the modest behavioral data currently available after radiation treatment, the reported peripheral and neuroinflammatory responses suggest that radiation can contribute to behavioral changes.

Other major gaps in understanding neurobiobehavioral changes in the context of cancer and cancer treatments pertain to the role of peripheral myeloid cells (monocytes, macrophages, and dendritic cells) and their potential localization to brain areas that interface with the peripheral circulation, such as the choroid plexus, perivascular spaces, and meninges. The chemokine CCL2 regulates myeloid cell infiltration (and potential inflammation) to different tissues, including these brain areas. For example, while CCL2 ablation in mice increases the peripheral pro-inflammatory cytokine response to LPS, it decreases the neuroinflammatory response in the entorhinal and frontal cortices and the hippocampus (139). Furthermore, CCL2 released by brain glioma tumors plays a key role in recruiting myeloid cells to the brain (140). However, the extent to which chemokine release by tumors in the periphery may influence immune cell trafficking to the brain and behavior remains unclear. On the other hand, CCL2 or peripheral immune cell trafficking to the brain may play a role in the context of chemotherapy-induced behavioral changes, as CCL2 ablation improves 5-FU chemotherapy-induced fatigue in tumor-free mice (141). A similar trafficking mechanism in spinal cord and DRG is also hypothesized to influence the development and persistence of CIPN (39, 42). The common neutropenia and lymphopenia side effects of chemotherapy may appear to conflict with the potential for increased innate immune cell trafficking to the brain and inflammation at first glance (142). When in fact, this immunogenic cell death results in production of DAMPs (proteins, nucleic acids, purines, and ROS), priming of CD4^+^ and CD8^+^ lymphocytes and a robust antigen-specific immune response against dead cell-associated antigens (143–145). Thus, cancer treatments overall consistently increase inflammation. These inflammatory mediators activate inflammasomes as well as TLRs to induce immunosurveillance or tumor progression (146), but also contribute to neuroinflammation, depression and neuropathic pain (147).

In summary, converging lines of evidence suggest that cancer and cancer treatments induce neuroinflammatory and behavioral changes in rodent models (Figure 1). Nevertheless, expansion of these initial basic science findings is required (Table 1). Specifically, the moderate variability in current microglial-related results from brain samples of models of tumors and cancer treatments necessitates a thorough temporal screening of brain microglial functioning and neuroinflammatory responses throughout tumor development and chemotherapy/radiotherapy treatments. This type of investigation would help to identify the cellular source/s of inflammation in the brain and elucidate the causal role of microglia in associated behavioral changes. Finally, several alternative pathways, including the sympathetic nervous system and the hypothalamic–pituitary–adrenal axis, modulate immune functions and, therefore, may be involved in the interaction among peripheral cancer, inflammation, and the brain.

### Link Between Neuroimmunology and the Neuroscience of Behavior

Support for neuroimmune signaling that is associated with changes in behavior is extensively reviewed elsewhere in the context of peripheral tumors alone (89) or chemotherapy (20, 23, 148). As previously discussed, peripheral inflammation due to cancer or chemotherapy can trigger microglial activation and associated neuroinflammation. In some reports, this neuroinflammation is associated with changes in neurons. For example, chronic cyclophosphamide chemotherapy treatment induces cognitive impairment, microglial activation, and impaired neuronal architecture (123). Attenuation of this neuroinflammation reverses the neural and behavioral changes, suggesting that the neuroinflammation preceded the structural changes to the neurons. Alternatively, chemotherapy may damage brain tissue directly, heralding in the inevitable local neuroinflammatory response. Indeed, chemotherapy induces brain cell death (e.g., *via* ROS production), synaptic damage, DAMP production, disruption of the BBB, mitochondrial dysfunction, white matter damage, and alterations in neurotransmitter availability (149, 150). For example, reports from multiple labs indicate that antimetabolite chemotherapy (5-fluorouracil; 5-FU) crosses the BBB thereby directly reducing myelination and neurogenesis, as well as disrupting learning and memory (27, 151, 152). In addition, intrahippocampal human neural stem cell treatment reverses the hippocampal microglial activation and impaired neuronal architecture, as well as cognitive impairments induced by chronic cyclophosphamide treatment in athymic nude rats (123), which suggests that the neuronal damage caused microglial activation. Regardless of the order of the events, converging evidence indicates that different inflammatory microenvironments can drive various microglia phenotypes that interact with CD4^+^ CD45^+^ cells to induce neuroprotection, neurodestruction, or unchanged neurobiology (153). While these direct microglial–neuron interactions have not yet been demonstrated in the context of cancer, other examples are available. Chronic stress-induced depressive-like behavior is mediated by an initial phase of microglial activation and proliferation followed by microglial apoptosis and suppressed hippocampal neurogenesis (154). Indeed, this dynamic microglial pattern may be similar to that over early and late periods of time after chemotherapy treatment. Furthermore, although microglia are most well-recognized for innate immune functions, increasing data indicate that non-pathological microglial functions are essential for normal brain development, as well as structural and functional processes in the adult CNS (155). In the healthy brain, microglia regulate the development and plasticity of neuronal circuit architecture, modulate synapse development, activity, and elimination, as well as modulate neurogenesis (156, 157). Thus, microglial activation under inflammatory conditions (potentially cancer or cancer treatments) also likely interferes with these basic neurobiological functions and thereby alters behavior.

Alternatively, microglia may indirectly affect neuronal function *via* astrocytes. Microglial activation has been shown to induce ATP release, which in turn stimulates purinergic receptors on astrocytes to modulate nearby neuronal electrophysiology (158). Although this work is *in vitro*, the interaction between the various glial cells and neurons constitute another putative mechanism for cancer-associated behavioral changes and an interesting area for future studies. Furthermore, a recent study indicates that the serotonergic pathways downstream of the serotonin (5-HT)_2B_ receptor in microglial cells contribute to neuronal synaptic refinement and brain maturation (159). These same 5-HT receptors can inhibit TLRs, thereby counteracting inflammation (160), and may, therefore, be a potential target to prevent cancer-associated behavioral changes. Indeed, an increasing number of studies suggest an involvement of the serotonergic system in the modulation of innate and adaptive immune functions (160–162).

## Cancer Patients

### Neuroimmunology and Cancer Treatments

For this section, the approach for finding original reports in the English language that considered neuroinflammatory factors and/or psychological and behavioral symptoms in cancer patients with tumors outside of the CNS before and after cancer treatment consisted of PubMed searches through April 2018 using combinations of the MeSH search terms: “depression,” or “anxiety,” or “cognition,” or “neuropsychological test”; “cancer,” or “tumor” or “chemotherapy” or “radiation”; “inflammation” or “imaging” or “microglia” or “brain.”

In the field of cancer research, it is well-accepted that affective disorders and cognitive impairments are more highly prevalent in cancer patients before, during and even years after cancer treatment relative to non-cancer controls (10, 163, 164). Although, cancer and cancer treatments share some potentially confounding physical symptoms (cachexia, fatigue, sleep disturbances) with major depressive disorder (MDD), meta-analyses and systematic reviews indicate that affective (MDD and “depressive”) and cognitive impairments are independent of these physical symptoms in cancer patients (165–168).

Although it has often been proposed that neuroinflammation may underlie the affective and cognitive deficits observed in cancer patients (23, 89, 169), scant neuroscientific data are attainable in patients. The most relevant clinical approach for understanding the relationship between neuroscience and cancer-associated behavioral comorbidities is neuroimaging. Of this neuroimaging work in cancer patients, studies focused on the effects of chemotherapy are most abundant, reviewed in Ref. (170). In the dominant breast cancer literature, cross-sectional neuroimaging approaches have yielded mostly consistent chemotherapy-induced deteriorations in neurostructure (using diffusion tensor imaging), some of which have been correlated with poor cognitive performance (171, 172). Specifically, cancer treatments reduce brain white and/or gray matter in the corpus callosum and cortex (173) or reduce hippocampal volume (171). These structural impairments are detectable over 20 years post-chemotherapy (174), and in fact, may be progressive (175, 176). Neuroinflammation is a top potential mechanism by which this occurs (177). Functional magnetic resonance imaging results (e.g., hippocampal activation during a cognitive task or at rest) are more mixed for chemotherapy-treated survivors (4, 178–181), perhaps due to the increased complexity of these assessments during active behavior. Neuroimaging cannot yet directly address the neuroinflammatory hypothesis; however, alterations in neuroimaging have been recently associated with peripheral inflammation in cancer patients treated with chemotherapy or radiation (182) and are associated with peripheral immune activation in other populations (183–186). Altered neuroimaging is also demonstrated in cancer patients prior to treatment, indicating that tumors outside of the brain influence brain network dynamics on their own (187, 188), possibly through immune signaling. For example, in breast cancer survivors at least 6 months after cancer treatment completion, peripheral inflammation is more strongly associated with amygdala reactivity to socially threatening images than in cancer-free controls (182).

Although less direct than neuroimaging, the positive association between cancer behavioral comorbidities and circulating inflammatory markers corroborates the neuroinflammatory theory and is well-supported (11, 58). In addition to baseline peripheral inflammatory markers, *in vitro* reactivity of peripheral immune cells is elevated in cancer patients with negative behavioral symptoms (189, 190), as are allelic profiles characterized by cytokine deregulation (191), and genetic polymorphisms of the inflammatory pathway [(192–194), but see Ref. (195)]. Furthermore, cytokine-based immunotherapy (IFN-α, IL-2 infusions) causes depression and cognitive impairments in cancer patients and other medically ill patients (196–198). Finally, there is a single neurobiological record of four adult (non-brain) cancer patients after high-dose chemotherapy treatment (199). Neuropathology is similar among these cancer patients and includes the loss of myelin and axons, as well as fluid and macrophage infiltration in various CNS regions. Similar brain pathology has been reported in autopsies of children with leukemia who were treated with chemotherapy (200). Taken together, these studies are consistent with the hypothesis that neuroimmune activation may be a key underlying mechanism of chemotherapy-induced cognitive impairment in cancer patients.

Many clinical studies that focus on chemotherapy effects on brain and behavior include cancer patients that also receive ionizing radiation therapy, although the individual role of radiation is rarely delineated. Thus, the effects of radiation therapy on neurobiology are poorly understood compared with chemotherapy. This oversight is relevant to many cancer patients; for example, radiation is used to treat approximately 56% of breast cancer patients (201). Abscopal effects, by which radiation used to treat a proximal tumor also reduces distal tumors, are thought to be immune-mediated (202). Specifically, dendritic cells and macrophages phagocytose cells damaged by radiation and then present tumor debris to adaptive immune cells to trigger widespread anti-tumor actions (203). As a result, circulating cytokines are elevated during radiation therapy in some cancer studies (204–206), but not others (207, 208). Breast cancer patients with higher baseline circulating inflammatory markers (C-reactive protein, myeloid-derived cells, IL-6) are also predisposed to fatigue after radiation (209). Thus, the potential for radiation-induced peripheral inflammation to potentiate neuroinflammation remains a viable hypothesis in need of further testing.

## Anti-Inflammatory Interventions (Pharmacological and Non-Pharmacological) in Rodents and Humans

To date, there are no standard clinical interventions for cancer behavioral comorbidities. Interventions used to reduce these behavioral issues by targeting inflammatory mechanisms include exercise, psychosocial interventions (210), and pharmacological anti-inflammatory treatments. Of note, pharmacological anti-inflammatory treatments have potential hematologic toxicity and cardiovascular side effects and may interact with other cancer treatments (211); therefore, greater emphasis has been placed on non-pharmacological interventions.

In breast cancer patients after chemotherapy, 12 weeks of hatha yoga improves self-reported cognitive function while reducing circulating inflammatory markers (212). Similar results were observed after 6-weeks of aerobic walking and resistance training (213) with the addition of increases in circulating anti-inflammatory markers. In the latter study, reductions in inflammation correlate with cognitive improvements. The same duration of lyengar yoga also reduces fatigue (214) while decreasing pro-inflammatory NF-κB activity (215). Furthermore, Qigong intervention (Chinese coordinated body posturing and movement) reduces circulating C-reactive protein as well as improves self-reported cognitive functioning (216).

In a subset of depressed cancer patients, 4 months of psychosocial intervention (relaxation and stress reduction exercises and education) improves mood while reducing inflammatory markers (217). Furthermore, cognitive–behavioral stress management intervention reduces pro-inflammatory gene expression of circulating immune cells from breast cancer patients, while decreasing negative affect and increasing positive affect relative to standard-of-care controls (218). However, other cognitive-based training that reduces depression and anxiety, increases inflammatory cytokine production in stimulated immune cells *in vitro* in breast cancer patients (219, 220). Finally, resistance-based exercise reduces radiation-induced increases in circulating pro-inflammatory cytokines, which mediates slight improvements in fatigue and pain (205). In another breast cancer subpopulation, characterized by mild to moderate depression and pain, a nonsteroidal anti-inflammatory drug that specifically inhibits COX-2 (celecoxib) improves depressive symptoms better than a non-selective COX inhibitor (211). Drugs that interfere with TNF-α signaling also improve fatigue in chemotherapy-treated cancer patients (221, 222).

In rodent models, similar interventions to reduce cancer treatment side effects include exercise and pharmacological anti-inflammatory treatments. Several studies indicate that voluntary (223) or forced (224, 225) aerobic exercise prevent cognitive impairments in chemotherapy-treated or brain-irradiated, tumor-free mice compared to sedentary control groups, while increasing hippocampal neurogenesis. Ibuprofen treatment reduces fatigue and depressive-like behaviors in tumor-bearing mice, while reducing IL-1β and IL-6 mRNA expression in the hippocampus, compared to healthy control mice (226). Furthermore, minocycline administration reduces central levels of pro-inflammatory cytokines and microglial activation, attenuating depressive-like behavior in tumor-bearing mice (55). Similarly, minocycline administration or functional blockade of a receptor expressed on myeloid cells attenuates cisplatin-induced CIPN (227) by suppressing the microglial pro-inflammatory response.

Alternative interventions include plant-derived adjuvant therapy drugs, such as those used in traditional Ayurvedic medicine (228). For example, pretreatment with rutin, astaxantin, or catechin significantly prevents the behavioral and neurobiological impairments induced by doxorubicin treatment in rodents (229–231). These bioceuticals also decrease TNF-α, prostaglandin E2, and COX-2 levels in hippocampus (230, 231). Furthermore, tetrahydrocurcumin exerts neuroprotective effects for vincristine-induced CIPN by decreasing oxidative stress, calcium and TNF-α levels in rats (232). These studies demonstrate the immunomodulatory, anti-inflammatory, and neuroprotective properties of these plant-based drugs in the context of chemotherapy.

## Conclusion

The current review organizes and evaluates the evidence supporting how cancer and cancer treatments can influence neuroimmune pathways, leading to behavioral and neurobiological changes. Notable progress has been made in cancer diagnoses and treatment, prioritizing the need for understanding and intervention that addresses the mental welfare of cancer survivors. Additional basic science research using various modeling approaches is required to untangle and to understand the interactions among various cancer treatments and their paradigms, tumor biology, and stress. These models will be essential to determining the role of neuroimmune pathways in neuronal and behavioral consequences of cancer. Complementary neuroimmune-focused information is warranted in clinical research, potentially *via* postmortem brain autopsies, magnetic resonance spectroscopy, and further studies of anti-inflammatory interventions. Finally, the extent to which cancer-induced behavioral changes differ from the same changes in other disease contexts can contribute to the understanding of factors that influence onset versus persistence of these comorbidities in cancer patients and survivors. In summary, increasing recent basic and clinical science evidence points to potentially additive neuroimmune mechanisms due to various components of the cancer experience in cancer-associated behavioral comorbidities (depression, anxiety, fatigue, cognitive disturbances, and neuropathic pain).

## Author Contributions

The authors contributed equally for conceptualization, data synthesis, and manuscript preparation of this review.

## Conflict of Interest Statement

The authors declare that the research was conducted in the absence of any commercial or financial relationships that could be construed as a potential conflict of interest. The handling Editor declared a shared affiliation, though no other collaboration, with one of the authors LP.
